# Factors Related to Psychological Distress in Suicide Prevention Supporters during the COVID-19 Pandemic

**DOI:** 10.3390/ijerph20064991

**Published:** 2023-03-12

**Authors:** Masana Ujihara, Hirokazu Tachikawa, Asumi Takahashi, Towa Gen, Yoshinori Cho

**Affiliations:** 1 Graduate School of Comprehensive Human Sciences, University of Tsukuba, Tsukuba 305-8575, Japan; 2 College of Nursing and Nutrition, Shukutoku University, Chiba 260-8703, Japan; 3 Department of Disaster and Community Psychiatry, Institute of Medicine, University of Tsukuba, Tsukuba 305-8575, Japan; 4 School of Humanities, Hokusei Gakuen University, Sapporo 004-8631, Japan; 5 Department of Psychiatry, Nanao Hospital, Tokyo 191-0055, Japan; 6 Department of Psychiatry, Teikyo University, Tokyo 173-8606, Japan

**Keywords:** suicide prevention, healthcare worker, helpline volunteer, psychological distress, COVID-19

## Abstract

Purpose: Psychological distress and related factors in suicide prevention supporters during the COVID-19 pandemic were clarified. Methods: A web-based survey for supporters from helplines or psychiatric institutions was conducted from May to July 2021. It included items about profession, stress and anxiety, and the Kessler Psychological Distress Scale. Results: 818 participants were analyzed. Psychological distress was significantly higher among healthcare workers in psychiatric institutions than among helpline volunteers. The factor most related to psychological distress in both professions was insufficient rest with overwork. Distress in helpline volunteers was related to their lack of ability to support people with suicidal thoughts and suicide attempts, excessive media coverage related to COVID-19, and trouble dealing with complainers. Distress in healthcare workers was related to their lack of ability to provide sufficient support to their clients due to infection prevention measures. Conclusion: Psychological distress among suicide prevention supporters during the pandemic has been affected by overwork, the fact that helpline volunteers cannot be trained in suicide prevention, and the fact that healthcare workers can only provide insufficient support to their clients due to infection prevention measures. To maintain suicide prevention during pandemics, it is necessary to implement measures that are tailored to the factors of psychological distress in supporters.

## 1. Introduction

The increased risk of suicide due to the COVID-19 pandemic is a great concern [1,2]. The number of suicides in Japan increased by 4.5% between 2019 (20,169) and 2020 (21,081), with a serious increase among women and young people [3,4]. Various changes in work, family, and social lives, such as increases in school closures, work-at-home jobs and caregiver roles, as well as limited access to healthcare services, may affect the risk of suicide by, for example, exacerbating family discord, creating childcare problems, and worsening caregiver fatigue [5]. We are cautioned not to discuss suicide and COVID-19 in easy terms [6], but in Japan, suicide prevention during the COVID-19 pandemic is a current and important issue.

Suicide prevention supporters (SPS), such as healthcare workers in psychiatric institutions (HCWs) and helpline volunteers (HVs), are always under severe pressure due to their heavy responsibilities. Damage to their own mental health is also a serious problem that must be cared for. HCWs are generally responsible for endeavoring to provide support under highly stressful conditions, such as having to deal with difficult mental health cases or being unable to prevent suicide [7]. Nurses who have experienced patient suicide often suffer grief similar to that of the patient’s bereaved family, but the support systems available to them are often limited to individualized support such as talking to their supervisor [8]. When HVs are dealing with a caller, they must be extremely careful about choosing their words, it is assumed that they experience a great deal of mental strain. Normally, about 14% of callers have suicidal ideations and 6% have a history of attempted suicide [9]. Contact with callers may be brief and anonymous, with little scope for caller feedback or follow-up [10], making it difficult to verify the effectiveness of their efforts, which may also be a factor in their sense of psychological distress.

During the COVID-19 pandemic, many studies have suggested that the mental health of healthcare workers has become an issue [11,12]. However, such studies have mainly focused on the mental health of frontline workers who directly respond to COVID-19-positive patients. These problems also affect psychiatric institutions and helplines in ways such as decreases in staff due to infection prevention, increases in consultation work due to infection fears, etc. [13]. So, SPSs are also affected by COVID-19 [14], but we have not found any studies that reveal the mental state of SPSs during a pandemic.

The COVID-19 pandemic is a worldwide disaster; however, the circumstances surrounding it in each country have differed. Increments in the number of suicides also differ. In Japan, the number of suicides did not increase; in fact, it actually decreased at the beginning of the pandemic. However, the suicide rate increased remarkedly after October 2020, especially among women and young people [15]. Unlike other countries, Japan’s infection countermeasures have not been based on government orders but rather on requests by the government, both national and local, for compliance. Because of the highly disciplined nature of Japanese culture, the population’s initial actions in response to the pandemic were quite acute, to the extent that people who refused to follow government-promoted anti-infection measures experienced extreme social pressure to comply. Because of the government’s early request to refrain from travelling across prefectural borders, even reasonable short-distance trips were disallowed for the average citizen. Moreover, daily trips to workplaces became difficult for many people, especially those relying on railways. These conditions surely affected their activities of SPSs.

Although the mental health of many people has deteriorated due to COVID-19, mental health resources are declining due to infection countermeasures [16]. With an increase in people with suicidal thoughts and limitations on their usual support resources due to infection control measures, the psychological distress experienced by SPSs is also likely to increase. The worsening mental health of SPSs may affect suicide prevention measures as their own attitudes toward suicide shift [17]. If the mental health of SPSs is not maintained, the efficacy of suicide prevention services may shrink gradually, making it crucial to consider measures for maintaining their mental health.

For the purpose of dealing with these problems, we conducted a large-scale web survey of SPSs to clarify their psychological distress and related factors during COVID-19 in order to provide suggestions for maintaining suicide prevention support systems.

## 2. Theoretical Framework

Many papers suggest that psychological distress among healthcare workers and personal assistance workers is adversely affected by the COVID-19 pandemic. Vizheh found that the psychological distress of healthcare workers is adversely affected by the COVID-19 pandemic [18]. Spoorthy found that the psychological distress faced by healthcare workers is associated with socio-demographic variables such as gender, profession, age, place of work, and department of work [19]. Muller found that HCWs reported anxiety, depression, sleep problems, and distress during the COVID-19 pandemic [20]. Heath found that there is a lack of literature on interventions for supporting the psychological well-being of healthcare workers during disease outbreaks [21]. These papers suggest that COVID-19 is producing negative mental health consequences for healthcare workers and personal assistance workers. Indeed, reduced technological support and increased workloads due to their own workplaces’ infection prevention measures are expected to be the biggest factors contributing to declines in well-being. We hypothesize that these deteriorating mental health conditions are also occurring in SPSs who devote themselves to the difficult task of suicide prevention.

SPSs in Japan can be divided into two main categories: indirect support through helplines and direct support through psychiatric health and welfare professionals (doctors, nurses, clinical psychologists, caseworkers, public health nurses, etc.). Since private organizations dedicated to mental health are still in their infancy, these professions can be said to represent Japan’s SPSs. In fact, the majority of the members of the Japanese Association for Suicide Prevention (JASP) [22], of which two of the authors serve as president and director, are also from the professions listed above.

One of the leading helpline organizations in Japan is “Inochi no Denwa” [23]. Its activities originated from telephone counseling for suicide prevention, which started in London, England in 1953. In Japan, the organization was led by a German missionary, Ms. Lutz Hettkamp, and in October 1971, Japan’s first telephone counseling service by volunteer counselors was launched in Tokyo. In 1977, there were only five Inochi no Denwa centers in Japan, and an organization was needed to play a central role in the nationwide development of this citizens’ movement. As of 2022, the number of member centers of the federation is 50, with approximately 5800 counselors. HVs are volunteers who have undergone six months of systematic training to acquire knowledge on suicide prevention.

In Japanese psychiatry, the main focus is on the treatment of psychiatric disorders underlying suicide, and HCWs at psychiatric hospitals and clinics provide patients with general counseling on suicide. When suicide attempts occur, corrective treatment such as hospitalization for medical care and protection is relatively common, as in other countries [24,25,26]. For this reason, psychiatry routinely treats patients with suicidal ideation through inpatient and outpatient treatment.

Therefore, the purpose of this survey was to clarify the psychological distress of SPSs during the COVID-19 pandemic, focusing on HVs and HCWs. These two categories are working under the leadership of JASP and, thus, are representative of Japanese SPSs.

## 3. Materials & Methods

### 3.1. Background and Recruitment Procedure

The survey was conducted using Survey Monkey [27] from May 26 to 12 July 2021. Around that period, the fourth surge of the pandemic hit Japan. About a hundred deaths and about five thousand new infection cases were counted per day at the peak of the surge. The government repeated declarations and reinstated the state-of-emergency in some areas. The use of remote work was spreading; however, such use of technology was still mostly limited to major corporations, and many people were still unable to use such technology. Vaccinations for COVID-19 had just begun about two months earlier, and most people only had their first dose. Although the number of deaths was considerably smaller than that in other countries, the sense of terror among people was still strong. “Hope” was not yet seen at that time [28].

In this environment, participants were recruited through the Japan Psychiatric Hospitals Association (1185 members), the Japanese Association of Neuro-Psychiatric Clinics (1639 members), and Inochi no Denwa. We asked each institution’s representatives to send flyers with a request letter and the URL for accessing the web-based survey to their members. 

Once participants provided informed consent online, the next survey pages were presented. Their answers were anonymous. No identifying information was collected, and no exclusion criteria were established. This study was approved by the Teikyo University Ethical Review Board for Medical and Health Research Involving Human Subjects (Registration No. 21-012) and the medical ethics committee of the University of Tsukuba (No. 1668-1).

### 3.2. Questionnaire

The questionnaire consisted of (1) demographics (gender, age, profession, years of experience in mental health welfare fields, existence of co-inhabitants), (2) experience with providing support for people with suicidal thoughts or who have made suicide attempts (since February 2020) and the approximate number of such individuals, (3) experience with providing support directly to COVID-19 (or suspected COVID-19) patients, (4) levels of stress and anxiety when providing care for suicidal individuals during COVID-19, and (5) the Kessler Six-Item Psychological Distress Scale (K6). 

We defined people with suicidal thoughts or suicide attempts as those who wanted to die by suicide or have attempted self-damaging behaviors with the intention of dying, specifically those triggered by issues caused by COVID-19 (e.g., unemployment). 

To determine the questionnaire items for stress and anxiety, the authors that are physicians and a public health nurse with expertise in psychiatric care and suicide prevention discussed the items based on an overview of previous studies [29,30,31] and challenges felt in clinical settings. Ultimately, 14 items and 1 open-ended question were decided upon. The items are shown in Table 1. 

K6 is a six-item short-version questionnaire developed to screen for mood disorders and anxiety disorders [32]. It assesses mood and anxiety experienced during the past month on a five-point scale, with a total score ranging from 0 to 24. We adopted the Japanese version, the reliability and validity of which has been confirmed [33]. K6 has a cutoff point for each level: psychological distress equivalent group (5–8), mood/anxiety disorder group (9–12), and serious mental illness group (13≤) [34]. For statistical analysis, this study applied a cutoff point of five to define two levels of psychological distress, since the participants included non-clinical samples and the purpose of the study was to explore a wide range of trends in mental distress.

This study was conducted under the auspices of JASP. The researchers, all of whom are well-versed in suicide prevention and serve on the board of JASP, reached a consensus on the appropriateness of the questionnaire items.

### 3.3. Statistical Analysis

SPSs were divided into HCWs, who mainly provide in-person treatment, and HVs, who mainly provide support by telephone. First, a simple tabulation was performed on the demographics of the respondents. Then, an χ-square test was conducted to assess differences in demographics and psychological distress between HVs and HCWs. Next, an analysis was conducted to determine the anxieties and worries associated with psychological distress in SPSs during the COVID-19 pandemic. Specifically, to clarify the comprehensive relationship between K6 scores (above or below the cutoff) and each item, odds ratios (OR) and 95% confidence intervals (CI) were calculated through a binomial logistic regression analysis using the forced entry method; the independent variable was levels of stress and anxiety when providing care for suicidal individuals during COVID-19, and the K6 score was the objective variable for each SPS. The analysis was performed on all data, except those with missing values, using IBM SPSS version 28.0.

## 4. Results

### 4.1. Characteristics of Respondents

All 1444 people began the survey, and a total of 818 respondents completed the full questionnaire (response rate 56.6%). Many participants did not respond to all questions, either stopping in the middle of the survey or closing the page. 

Table 1 shows the demographics and characteristics of the respondents. Compared to HCW respondents, there were significantly more female than male HVs, more who were in their 60s or older, and more who had greater experience being in contact with people with suicidal thoughts or who had made suicide attempts. However, significantly more HCWs had over 10 years of experience in the mental health welfare field, had treated COVID-19-positive or suspected positive patients, and had K6 scores of five or higher.

Table 2 shows the numbers and percentages of responses to the stress and anxiety items by SPSs. HVs answered “Lack of ability to support people with suicidal thoughts and suicide attempts” (15.8%), “Lack of information on available social resources” (14.1%), and “Excessive media coverage related to COVID-19” (12.7%) more frequently than HCWs did. HCWs answered the following more often than HVs: “Danger of being infected with COVID-19” (22.3%), “Lack of ability to deal with COVID-19′’ (14.3%), and “Unable to provide sufficient support due to infection prevention measures” (10.5%).

### 4.2. Relationship between Psychological Distress and Each Item

Table 3 shows that, for the HVs, the following four items were significantly related to K6 cutoff points: ”Excessive media coverage related to COVID-19” (OR = 4.81, 95% CI = 1.30–17.79, *p* = 0.019), “Trouble dealing with complainers (those who make excessive demands)” (OR = 5.75, 95% CI = 1.38–23.97, *p* = 0.016), “Insufficient rest with overwork” (OR = 16.52, 95% CI = 3.98–68.54, *p* < 0.001), and “Lack of ability to support people with suicidal thoughts and suicide attempts’’ (OR = 8.37, 95% CI = 1.42–49.30, *p* = 0.019). As for HCWs, two items were significantly related to K6 cutoff points: “Unable to provide sufficient support due to infection prevention measures” (OR = 1.99, 95% CI = 1.11–3.58, *p* = 0.022) and “Insufficient rest with overwork” (OR = 7.57, 95% CI = 2.94–19.51, *p* < 0.001). “Insufficient rest with overwork” had the highest odds ratio in both SPS groups. The Nagelkerke R-square value, which is an indicator of the goodness of fit of the model, was confirmed to be 0.370 and 0.249 for the HVs and HCWs groups, respectively. 

### 4.3. Special Note about the Open-Ended Question

Since this paper is primarily a quantitative analysis, the analysis of the open-ended question will be reported separately. However, some points that strengthen the argument of this paper should be noted here. 

Several HV responses point to problems with the attitude of the mass media:

- Media reports stirred up anxiety about COVID-19 in order to draw attention to the issue.
- The impact of the celebrity suicides, which were widely reported on around the same time.
- The problematic nature of how the Inochi no Denwa was being depicted by the media at that time.
- Anger and helplessness over these issues.

HVs pointed out the problem that the daily reports of the number of people infected with COVID-19 and the number of deaths increased the anxiety of suicidal ideologues. Unfortunately, Japan had a series of famous actors and actresses die, probably due to suicide, in 2020, a year when the COVID-19 pandemic was severe. Many of the HVs indicated that the content of news reports clearly communicated the means of suicide, and the fact that the reports were solely about the Inochi no Denwa as a consultation center led to anger and a sense of helplessness. In addition, there were many descriptions of the lack of government support measures. In particular, HVs pointed to the inadequacy or lack of financial support for those who lost their jobs or had to close their businesses because of the COVID-19 pandemic. 

HCWs often described the following:

- Insufficient understanding of infection control measures for patients
- Insufficient telemedicine system

Patients refused to wear masks, and supporters voiced concerns about the lack of protective clothing, etc. In addition, Japanese hospitals and clinics had few or no telemedicine systems in place prior to the COVID-19 pandemic, so some complained about this. Although not many, some HCWs have reported difficulty in maintaining their own mental health due to the self-restricting lifestyle they were leading at that time, which limited their ability to cope with stress as they normally would.

## 5. Discussion

### 5.1. Comparison of HVs and HCWs

Table 1 shows considerable differences between the characteristics of HVs and HCWs. HVs were more likely to be female and aged 60 or older, which is much older than the ages of HCWs. This is largely due to the fact that HVs work as volunteers. In Japan, women have more opportunities to engage with others as volunteers than men. In addition, HVs are older in age because many of them started their volunteer activities after retirement. This is also why HVs have fewer years of experience in the field of mental health welfare compared to HCWs. It was assumed at first that HVs, many of whom are elderly, are at a high risk of severe infection by COVID-19 and therefore have strong infection anxiety. However, the proportion of HCWs who answered “yes” to the question “Danger of being infected with COVID-19” was higher. This may be attributed to the fact that HVs were consulted remotely by telephone and had fewer opportunities to directly interact with the target population. In addition, as noted in the open-ended question responses, it is presumed that this is also because, at the time of the survey, there were problems such as inadequate infection control measures in hospitals and clinics and patients’ refusal to wear masks.

The number of people treated was higher for HVs, presumably because of the constant telephone consultations. Conversely, COVID-19-related treatment was higher among HCWs, as they were more likely to be involved in more concentrated contact with infected individuals due to their direct support. Overall, the finding that HVs also had better mental health is an important point of this paper. The fact that the HVs provided indirect support and had less opportunity to directly engage with COVID-19-related patients and the fact that they were volunteers may have made a difference in terms of responsibility compared to healthcare professionals.

In both groups, many answered “yes” to the items “Danger of being infected with COVID-19,” “Poor tele-support technology,” Lack of ability to deal with COVID-19,” and “Lack of information on available social resources”. However, the impact of these items on their psychological distress was small, suggesting that these concerns were not a serious source of worry for most people. In other words, these items have been felt by many people during the COVID-19 pandemic, but their impact on psychological distress has been small.

### 5.2. Psychological Distress of HVs

The survey clearly showed the severity of the psychological distress among SPSs, who were aware of their excessive workloads. Factors influencing psychological distress differed between professions. In particular, among HVs, while only a few people answered “yes” to being overworked, its contribution to their psychological distress was high, which is to be expected in a situation where some people with a high sense of mission and responsibility are trying too hard. Among HCWs, however, while more people answered “yes” to being overworked, its impact on their psychological distress was lower. Based on these results, it can be seen that some HVs are seriously overworked, albeit infrequently, and that measures to find and remedy such situations are needed. Of the 13 helpline offices throughout Japan, 25% were forced to temporarily suspend consultations, or, for example, an office with two staff members often became a one-person office due to COVID-19. Due to such closures and cutbacks, some incoming calls were not responded to [35]. In addition, as mentioned among the open-ended question responses, the mass media repeatedly reported on the “Inochi no Denwa” as a consultation center for suicide prevention, which probably contributed to the increase in the number of calls.

Psychological distress was significantly related to a lack of an ability to support persons at a high risk of suicide. HVs go through thorough, systematic training before beginning their volunteer activities, and they participate in monthly group study sessions during their internship period [36]. However, such training could not be conducted during COVID-19; thus, their anxiety about not being able to maintain their knowledge and skills might have led to psychological distress. Furthermore, some two-person shifts were reduced to one-person shifts due to infection prevention measures, making it impossible to provide adequate peer support [37]. An effective countermeasure may be for the local government to collaborate in securing larger office spaces, take measures to reduce the risk of infection, and allow more than two HVs to work at a site at the same time. Furthermore, the current results indicating that HVs, but not HCWs, were affected by COVID-19-related media coverage suggest that the daily COVID-19 news coverage affected the mental health of HVs by mediating the nature of callers’ consultations. Additionally, excessive news coverage could exacerbate the mental health of HVs who are already in a vulnerable condition. It is necessary to understand these situations correctly and take countermeasures.

There remain some unresolved issues: The implications of the results regarding the psychological training provided to HVs and regarding the need to provide supervision groups are issues for future research.

### 5.3. Psychological Distress of HCWs

This survey shows that HCWs had a relatively high rate of overwork. We speculate that this is not only due to the increased need for suicide prevention care but also to the insufficient limiting of clinic hours and the inadequate space for infection prevention, which may have led to overwork and negatively affected their mental health. Organizations need to examine the nature of their work environments regarding overwork and promptly implement management measures, such as securing sufficient space and personnel to ensure adequate rest periods.

It was found that HCWs were significantly more likely to report insufficient treatment, counseling, and care due to infection prevention measures, suggesting that they face the dilemma of choosing between being hindered in providing regular support due to infection control measures and placing themselves at risk. The inability to provide proper care is thought to cause moral distress to HCWs, especially when it involves a life-or-death task such as suicide prevention. Moral distress has been reported in healthcare settings such as ICUs, where patients often die [38]. During the COVID-19 pandemic, there has been additional recognition of the moral injury resulting from the inability to provide dignified end-of-life care due to infection control measures and the prioritization of ventilator placement [29]. This study suggests that these situations also extend to healthcare workers in psychiatry.

To resolve these issues, it is important for organizations to ensure the facilitation of debriefs and moral building communal time and to encourage two-way dialogue with openness to suggestions and ideas from staff. Additionally, the enhancement of online medical care systems seems to be an effective measure for maintaining the same quality of care as in ordinary times. In particular, efforts must be made to create an environment in which sufficient support can be provided to patients, even in unusual situations such as infection control, to prevent HCWs from experiencing moral distress. How to apply these methods to SPSs in general is an issue for future study. To prevent the shrinkage of local resources for suicide prevention during the COVID-19 pandemic, it is necessary to implement factor-specific countermeasures.

## 6. Limitations

This study targeted only Japanese SPSs, and the context of psychological distress may differ in other countries due to cultural and institutional backgrounds, survey timing, and COVID-19 infection rates, so it is difficult to generalize the current results. In addition, the use of convenience samples, the use of untested questionnaires with respect to items that cause stress and anxiety, and the high rate of questionnaire non-completion should be taken into account. Furthermore, it is possible that factors not examined in this survey—for example, being able to take the time to respond to the survey or the condition of participants’ residential areas, may be related to psychological distress. Finally, this study focused on two professions, but the term suicide prevention supporter includes a broader range of professions. Target professions in future research should be broadened and examined for different factors affecting psychological distress.

## 7. Conclusions

Psychological distress and related factors in suicide prevention supporters during the COVID-19 pandemic were clarified. As the COVID-19 pandemic persists, with no end in sight, this is the first research work that has clarified some of the factors related to psychological distress in SPSs. In addition to overwork, the factors that most detrimentally affect HVs are not having access to training in suicide prevention, excessive media coverage about COVID-19, and trouble dealing with complainers, and the factor that is most detrimental to HCWs is providing insufficient support to their clients due to infection prevention measures. To prevent the shrinkage of local resources for suicide prevention during pandemics, it is necessary to implement measures that are tailored to the factors of psychological distress in SPSs.

## Figures and Tables

**Table 1:** Characteristics of SPSs (*n* = 818).

Variables	*N*	%	HV (*N* = 537)		HCW (*N* = 281)		*p* Value
			*N*	%	*N*	%	
Sex							<0.001
Male	536	65.5	145	27	137	48.8	
Female	282	34.5	392	73	144	51.2	
Age group							<0.001
20–59	380	46.5	177	33	203	72.2	
20 s	18	2.2	1	0.2	17	6	
30 s	42	5.1	7	1.3	35	12.5	
40 s	106	13	31	5.8	75	26.7	
50 s	214	26.2	138	25.7	76	27	
≥60	438	53.5	360	67	78	27.8	
60 s	304	37.2	240	44.7	64	22.8	
≥70	134	16.4	120	22.3	14	5	
Co-inhabitants							0.462
Yes	667	81.5	434	80.8	233	82.9	
No	151	18.5	103	19.2	48	17.1	
Years’ experience in mental health welfare field							<0.001
0–9	411	50.2	321	59.8	90	32.03	
<1	85	10.4	67	12.5	18	6.4	
1–3	101	12.3	82	15.3	19	6.8	
4–6	127	15.5	104	19.4	23	8.2	
7–9	98	12	68	12.7	30	10.7	
10-	407	49.8	216	40.2	191	68	
10–20	210	25.7	143	26.6	67	23.8	
≥21	197	24.1	73	13.6	124	44.1	
Treatment of people who had suicidal thoughts or who had attempted suicide triggered by the COVID-19 pandemic (from February 2020)							<0.001
Yes	535	65.4	412	76.7	123	43.8	
No	283	34.5	125	23.3	158	56.2	
Number of people treated							<0.001
0–3	610	74.6	377	70.2	233	82.9	
4-	208	25.4	160	29.8	48	17.1	
4–6	115	14.1	86	16	29	10.3	
7–9	34	4.2	25	4.7	9	3.2	
10–30	47	5.7	39	7.3	8	2.8	
≥31	12	1.5	10	1.9	2	0.7	
COVID-19-related treatment							<0.001
Yes	158	19.3	45	8.4	113	40.2	
No	660	80.7	492	91.6	168	59.8	
K6 score							<0.001
0–4	543	66.4	379	70.6	164	58.4	
5-	275	33.6	158	29.4	117	41.6	

Note. *p*-values are by binary comparison via an x-square test. HV: Helpline volunteers, HCW: Healthcare workers.

**Table 2:** Stress and Anxiety When Providing Care for Suicidal Individuals During the COVID-19 Pandemic (*n* = 818).

Variables	HV		HCW	
	*N*	%	*N*	%
Danger of being infected with COVID-19	180	17.8	167	22.3
Unable to provide sufficient support due to infection prevention measures	69	6.8	79	10.5
Poor tele-support technology	47	4.6	78	10.4
Slandered, discriminated against, or bullied	4	0.4	11	1.5
Poor infection control in the workplace	26	2.6	34	4.5
Poor support from supervisors and co-workers at work	6	0.6	14	1.9
Trouble dealing with complainers (those who make excessive demands)	74	7.3	40	5.3
Accusations and harassment by clients	75	7.4	9	1.2
Insufficient rest with overwork	8	0.8	34	4.5
Lack of ability to deal with COVID-19	85	8.4	107	14.3
Lack of ability to support people with suicidal thoughts and suicide attempts	160	15.8	24	3.2
Lack of information on available social resources	143	14.1	57	7.6
Excessive media coverage related to COVID-19	129	12.7	77	10.3
Lack of support in their personal lives, including places to stay and childcare	8	0.8	18	2.4

Note: Multiple selection. HV: Helpline volunteers, HCW: Healthcare workers.

**Table 3:** Association Between Variables and Psychological Distress in Binomial Logistic Regression.

Variables	OR	[95% CI]	*p* Value
HV			
Insufficient rest with overwork	16.52	[3.98–68.54]	<0.001
Lack of ability to support people with suicidal thoughts and suicide attempts	8.37	[1.42–49.30]	0.019
Excessive media coverage related to COVID-19	4.81	[1.30–17.79]	0.019
Trouble dealing with complainers (those who make excessive demands)	5.75	[1.38–23.97]	0.016
HCW			
Insufficient rest with overwork	7.57	[2.94–19.51]	<0.001
Unable to provide sufficient support to their clients due to infection prevention measures	1.99	[1.11–3.58]	0.022

Note. *p*-values reflect binomial logistic regression analysis with the forced entry method; K6 points are objective variables; all items are adjustment variables. HV: Helpline volunteers, HCW: Healthcare workers, OR: Odds ratio, CI: Confidence interval.

## Data Availability

Not applicable.
